# Light buckets and laser beams: mechanisms and applications of photobiomodulation (PBM) therapy

**DOI:** 10.1007/s11357-025-01505-z

**Published:** 2025-01-18

**Authors:** David W. Frankowski, Luigi Ferrucci, Praveen R. Arany, Dawn Bowers, Janis T. Eells, Francisco Gonzalez-Lima, Nicole L. Lohr, Brendan J. Quirk, Harry T. Whelan, Edward G. Lakatta

**Affiliations:** 1 National Institute On Aging, Bethesda, MD USA; 2 University at Buffalo, Buffalo, NY USA; 3 University of Florida, Gainesville, FL USA; 4 University of Wisconsin-Milwaukee, Milwaukee, WI USA; 5 The University of Texas at Austin, Austin, TX USA; 6 The University of Alabama at Birmingham, Birmingham, AL USA; 7 Medical College of Wisconsin, Milwaukee, WI USA

**Keywords:** Photobiomodulation therapy, Non-ionizing light therapy, Non-thermal light therapy, Mitochondrial cytochrome C oxidase (CcO), Transforming growth factor-β1 (TGF-β1), Near-infrared (NIR) Light, Aging and age-associated diseases, Neuroprotection, Therapeutic applications of light

## Abstract

Photobiomodulation (PBM) therapy, a non-thermal light therapy using nonionizing light sources, has shown therapeutic potential across diverse biological processes, including aging and age-associated diseases. In 2023, scientists from the National Institute on Aging (NIA) Intramural and Extramural programs convened a workshop on the topic of PBM to discuss various proposed mechanisms of PBM action, including the stimulation of mitochondrial cytochrome C oxidase, modulation of cell membrane transporters and receptors, and the activation of transforming growth factor-β1. They also reviewed potential therapeutic applications of PBM across a range of conditions, including cardiovascular disease, retinal disease, Parkinson’s disease, and cognitive impairment. Workshop participants largely agreed that PBM holds immense potential as a safe and effective therapeutic approach for a wide range of age-related diseases and cognitive decline. While further research is needed to fully elucidate its mechanisms and optimize treatment protocols, the findings presented at the NIA workshop provide strong evidence for the continued investigation and clinical translation of this promising, inexpensive, safe technology, to aging and age-associated diseases. Here, we review the research presented and discussion held at the meeting. In addition, the text has been updated, where applicable, with recent research findings that have been made since the meeting occurred.

## Introduction

On January 10, 2023, scientists from the National Institute on Aging (NIA) Intramural and Extramural programs convened a workshop on the topic of photobiomodulation (PBM). PBM is the process of applying low intensity light to tissue to produce physiological responses. The therapeutic effects of PBM have been documented across a wide variety of biological processes and, of particular interest to the NIA, it has been suggested that it may promote longevity and better health. However, the specific mechanisms of action of PBM are not entirely understood and the methodology of various light therapies is ill-defined. For this reason, experts convened to discuss the topic of PBM and its potential applications to aging and age-associated diseases, such as peripheral artery disease (PAD) and many others. Due to the relatively nascent status of the field, the goal of the workshop was to examine or present the commonalities and differences among light therapy types and indications, and to explore hypotheses about underlying mechanisms and existing gaps in knowledge. The final goal was to outline a path forward for advancing PBM as a safe and reliable intervention for age-associated diseases.

Light has the dual property of wavelength and particles, spanning sub-atomic to universe scales, with the ability to induce various molecular, cellular, and clinical responses. Unsurprisingly, then, light has been shown to possess therapeutic uses and has a long history in medicine, most notably in photothermal disinfection in chronic wounds and gum disease, with use of lasers in various surgical procedures involving hard and soft tissue [1]. However, non-surgical applications for light treatment, commonly known as photodynamic therapy (PDT) and photobiomodulation (PBM) therapy (as it is now known), have existed since the 1960s, when it was inadvertently discovered that exposure to low powered ruby laser light improved hair regrowth in mice [2].

PBM (MeSH term) is defined as a form of light therapy that uses nonionizing light sources (e.g., lasers, light emitting diode (LED) devices, and broad-spectrum lamps) to deliver light energy in the visible and near-infrared (NIR) portion of the electromagnetic spectrum with wavelengths between 400 and 1400 nm. PBM is a non-thermal process that engages several physiological mechanisms and elicits both primary and secondary effects that culminate in different cellular responses. Thus, PBM is considered to work at the cellular response levels to elicit its therapeutic effects.

Since the early work of Mester and colleagues, PBM has been shown to have therapeutic benefits in tissue repair, inflammation suppression, and pain relief [3]. Recent discoveries even suggest potential benefits of light therapy in the treatment of Alzheimer’s disease, Parkinson’s disease, multiple sclerosis, and osteoarthritis, to name a few [4–8]. However, most studies have been conducted in animal models. Thus, current and future work must build upon these animal models to translate PBM therapy from the laboratory to the clinic.

## Proposed mechanisms of PBM

**Fig. 1:**
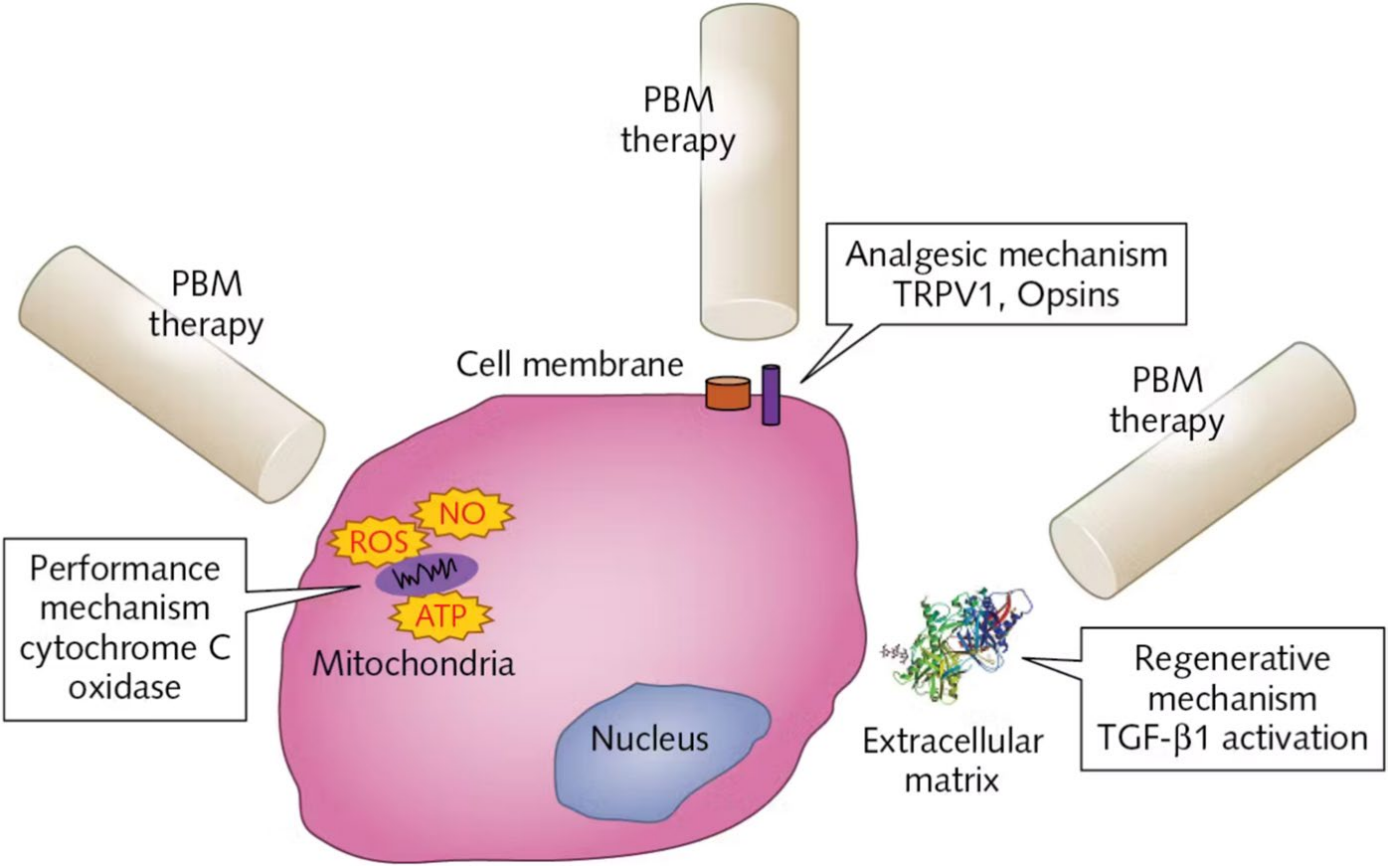
Three proposed mechanisms of action for PBM. (Adapted from P. Arany, 2019)

Three proposed mechanisms by which PBM may produce therapeutic effects were discussed at the workshop (Fig. 1). The first, and most investigated, entails the intracellular targeting of mitochondrial cytochrome C oxidase (CcO), an enzyme capable of absorbing red light. Effects on CcO have been shown to induce transient increases in ATP and ROS, which can have various downstream consequences. The second mechanism involves cell membrane transporters and receptors that are light sensitive, such as opsins, AHR, and TRPV1. Light absorption by these molecules can affect G-protein-coupled receptors (GPCRs) signaling and ion flux, including of calcium. The third involves the extracellular activation of transforming growth factor-β1 (TGF-β1), which can promote downstream responses that engage signaling molecules such as Smad, MAPK, NFκB, and ATF-4 and are involved in tissue fibrosis and intercellular matrix metabolism. Depending on cellular context and the surrounding environment, PBM has the potential to induce a diverse range of molecular and cellular outcomes.

### Mitochondrial cytochrome C oxidase (CcO)

CcO is a large, multi-subunit transmembrane protein found in the mitochondria of eukaryotes and in some aerobic bacteria. In the mitochondria, CcO catalyzes the respiratory reduction of oxygen to water and employs basic principles of redox-coupled proton pumping to establish a proton gradient across the inner-mitochondrial membrane for ATP production. Thus, CcO is critical for ATP production, and genetic mutations that alter CcO functionality or structure can result in severe, often fatal metabolic disorders.

**Fig. 2:**
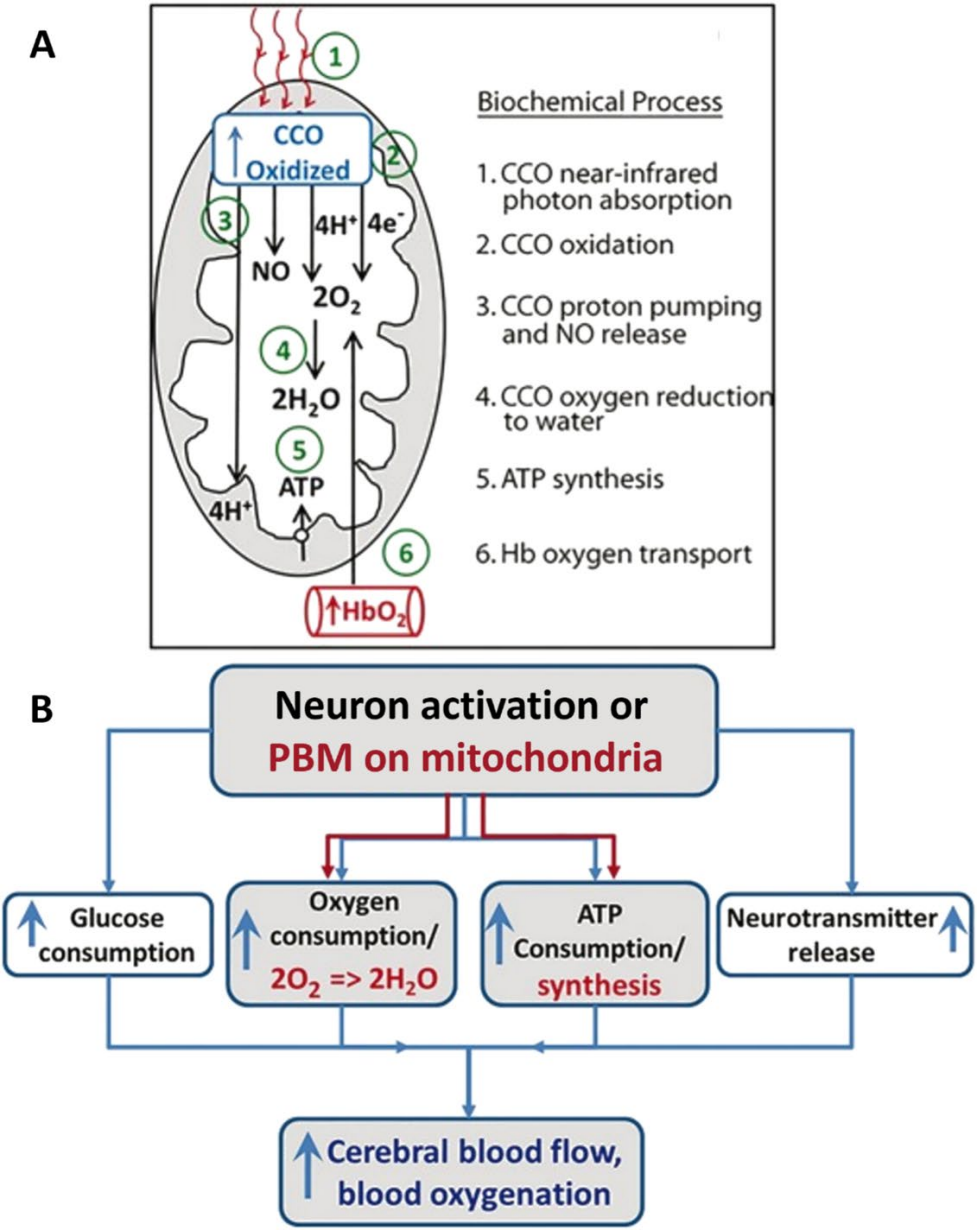
**A** Model of the photobiochemical mechanism of action of infrared light on the measured cytochrome c oxidase oxidation (CcO oxidized) and hemoglobin oxygenation (HbO_2_) during 1064-nm laser stimulation. **B** A flow chart illustrating the conventional neuro-vascular coupling by the black-colored notations and PBM-induced metabolic-hemodynamic coupling by the red-colored notations. The blue-colored notations represent common endpoints of both mechanisms on cerebral circulation. Adapted from Wang et al. (2017)

Most theories of PBM involve CcO as a key cellular target of red light or near infrared light (NIR) treatments. Evidence for the participation of CcO in PBM includes increased expression and activity of CcO as assessed by immunohistochemistry, histochemistry, and cell extract assays, as well as increased oxygen uptake by cells and mitochondria following light exposure [9]. Researchers posit that NIR irradiation of mitochondria removes electrons from CcO which serves as a catalyst for ATP production as well as the release of nitric oxide (NO) and reactive oxygen species (ROS), which ultimately increases the cerebrovascular delivery of oxygen to metabolically hungry tissue (Fig. 2; Wang et al., 2017). In addition, the dissociation of NO from its binding site on CcO may result in an enhancement of CcO activity and activation of transcription factors leading to changes in gene expression (10). This cascade of events triggered by NIR irradiation of CcO in the mitochondria appears to increase blood oxygenation and flow, upregulate antioxidant genes, increase expression of genes for cell proliferation, and decrease genes for pro-inflammatory proteins [11].

To gain insights into the underlying in vivo mechanisms of PBM, Gonzalez-Lima and colleagues determined the consequences of delivery of 1064-nm laser stimulation to the forehead of healthy participants on CcO oxidation and hemodynamic changes [12]. This transcranial PBM, which is estimated to penetrate up to 40 mm into the brain cortex [13], was found to induce up-regulation of oxidized CcO in the human brain in both younger and older people. Additional analysis revealed that PBM initiates a series of reactions linked to energy production inside mitochondria, which may be divided into six biochemical processes: CcO photon absorption, CcO oxidation, proton pumping and NO release, CcO-catalyzed oxygen reduction to water, ATP synthesis by oxidative phosphorylation, and coupled hemoglobin oxygen transport.

Extending the above work, Cardoso and colleagues employed quantitative CcO histochemistry to map brain region differences in CcO activity in healthy young (4 months old) and aged (20 months old) rats [14]. Control groups received sham stimulation, while treated groups were administered transcranial PBM (810 nm wavelength and 100 mW power) for 58 consecutive days. It was observed that with aging regional brain CcO activity was mostly weaker, as was the systems-level functional connectivity, particularly between the sensorimotor and limbic regions, and that chronic laser treatment reversed these age-related phenotypes. To further examine the effects of age on the mechanistic responses, PBM was administered using an FDA-approved class IV laser device to the right anterior prefrontal cortex of 68 healthy younger and older adults, ages 18–85, in a randomized, sham-controlled study [15]. Broadband near-infrared spectroscopy was used as a noninvasive method to quantify bilateral cortical changes in oxidized CcO and hemoglobin oxygenation before, during and after 1064-nm wavelength laser treatment. As compared to sham (and thermal) controls, a significant, rapid and persistent laser-induced increase in oxidized CcO was observed during and after laser administration, followed by a significant post-stimulation increase in oxygenated hemoglobin and a decrease in deoxygenated hemoglobin. Notably, there was a greater laser-induced effect on CcO with increasing age, though laser-induced effects on cerebral hemodynamics decreased with age. The collective findings indicate that PBM augments’ cerebral photo-oxidized CcO, alleviates the age-related decline in mitochondrial respiration, and promotes cerebrovascular oxygenation, substantiating further research on the therapeutic potential of PBM in cognitive enhancement and neuroprotection in neurologic and psychiatric diseases.

### Cell membrane transporters and receptors

A second mechanism involving PBM-modulated intra-membrane transport was mentioned only briefly, likely because research is still in a nascent phase but still worth noting. Of specific interest are the family of transient receptor potential (TRP) channels. These channels are photoreceptive, and it appears that exposure to light allows an influx of calcium through the TRP channel into the cell, that then affects downstream cellular processing [6]. Laser light (640 nm) was shown to act specifically on TRPV2 receptors, triggering an immune response in human mast cells and may have clinical implications involving analgesia [16]. Photostimulation of retinal ganglion cells have also been shown to trigger membrane potentials through activation of TRPV4 receptors that open sodium and calcium channels [17]. Further investigation of this mechanism and how it may be leveraged clinically may yield important discoveries.

### Transforming growth factor-β1 (TGF-β1)

**Fig. 3:**
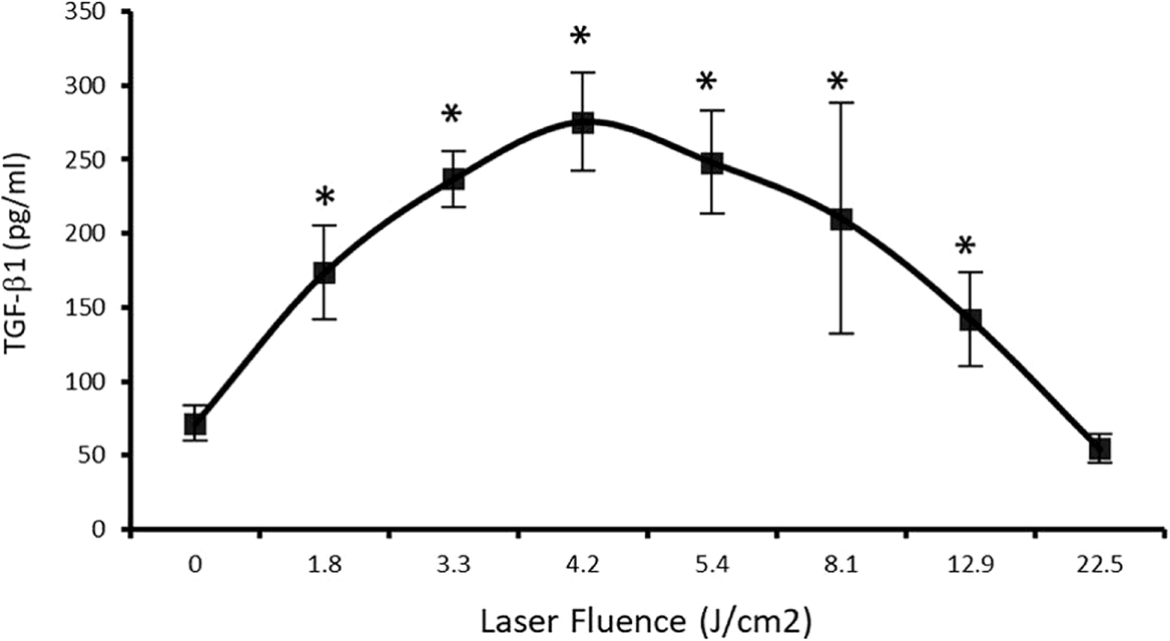
Biphasic PBM dose curve on levels of TGF-β1. Biphasic PBM dose curve showing activation of latent TGF-β1 in serum. TGF-b1 activation in serum after treatment with a low-power laser at increasing fluences assessed with ELISA. Adapted from Arany (2014)

A third PBM mechanism of interest at the meeting involved circulating extracellular transforming growth factor-β1 (TGF-β1), a cytokine that helps regulate immune responses [18] and is involved in regulating stem cell differentiation [19, 20]. Arany and colleagues have demonstrated that PBM treatment induces reactive oxygen species (ROS) in a dose-dependent manner (Fig. 3), which, in turn, activates a redox sensitive site within TGF-β1 to promote differentiation of host stem cells and support dental tissue regeneration [21]. Additional case studies show that PBM can alleviate oral tissue fibrosis, presumably via the TGF mechanism [22]. This mechanism may play an important role in future PBM therapy, as emerging studies have indicated potential therapeutic roles for TGF-β1 in not only various wound models, but in cancer immunotherapy, Alzheimer disease, and cardiovascular disease.

## PBM applications discussed

### Cardiovascular disease

**Fig. 4:**
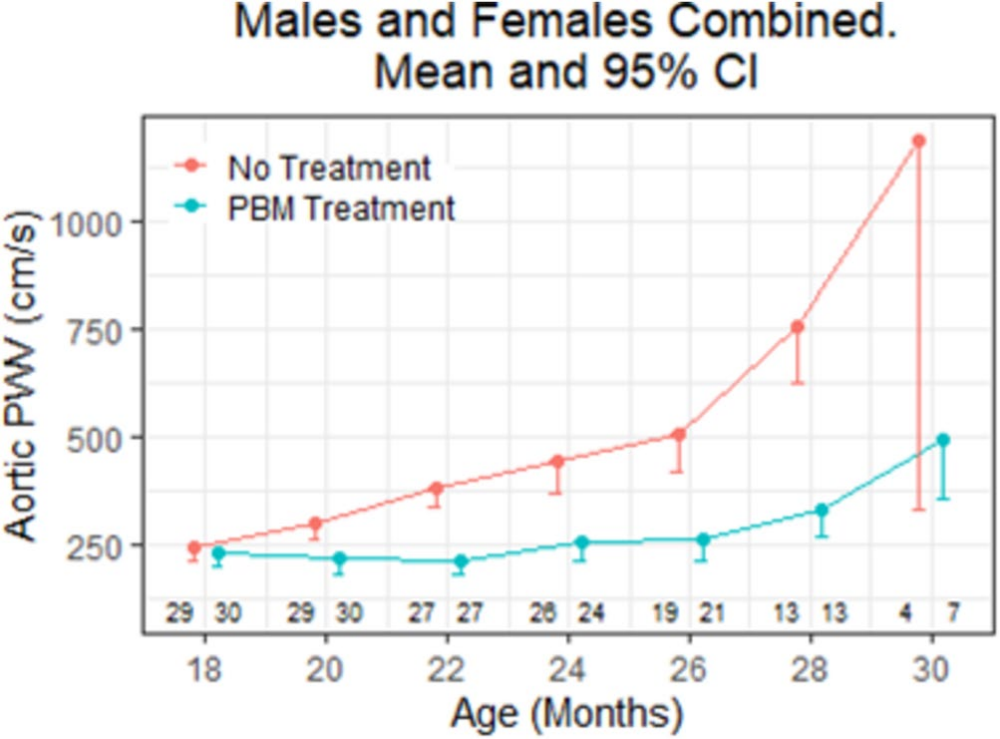
Effect of Age and PBM treatment on aortic stiffness assessed as aortic pulse wave velocity (PWV). Aged C57/BL6 mice. PBM-treated mice exposed to NIR light (3 J/cm.^2^, 2 min/d, 5 d/w; 850 nm). Timepoints around each age are offset for visualization purposes. Error bars indicate SD; numbers on the lower part of the figure represent the combined number of untreated and treated male and female mice at each age. Redrawn from data in Ahmet et al. (2023)

Much discussion centered around cardiovascular diseases. Aging hearts and arteries operate on the edge of cardiovascular disease. As one ages, there is the potential to cross the clinical practice threshold of hearts and arteries, associated with subclinical inflammation, resulting in the development of disease, such as hypertension, coronary ischemia, cerebral ischemia, and left ventricle (LV) hypertrophy. To address the limitations of past cross-sectional investigations of how advanced age affects cardiovascular functions, Lakatta and colleagues designed and recently executed a longitudinal study on C57/BL6 mice that assesses heartbeat interval variability at 3-month intervals, beginning at 6 months of age and continuing to the end of life [23]. The primary purpose of this study was to understand the deterioration of intrinsic sinoatrial node (SAN) function and compensatory autonomic signatures of heartbeat interval variability in advanced age [23]. However, Lakatta and colleagues also sought to determine the potential physiological benefits of photobiomodulation (PBM) in male and female mice relative to a comparable untreated control group [24]. Near infrared (NIR) therapy (3 J/cm^2^, 2 min/d, 5 d/w; 850 nm) was administered from 18 months of age, examining each mouse every 2 months until death for echocardiographic assessment of LV structure and function, cardiac function, and arterial structure/function, as well as gait speed and frailty index. A complementary cross-sectional study was carried out for the purpose of isolating tissue samples following sacrifice at different ages. Lakatta and colleagues found that PBM treatment had a statistically significant effect on left ventricular wall thickness (LVWT) and left atrial dimension (LAD). In addition, PBM treatment beneficially affected aortic diameter (AoD), thoracic aorta (TA), distensibility, and pulse wave velocity (PWV; Fig. 4); interactions between age and treatment were observed for the latter two. Finally, there was a significant effect of age and PBM treatment, as well as an age-treatment interaction, on the measured frailty index (a compilation of 31 parameters). In short, chronic PBM therapy appeared to ameliorate heart and arterial structure/function deterioration and reduced the age-associated increase in frailty index.

In a related study, Lakatta and colleagues examined the effects of PBM therapy on age-associated cardiovascular changes in a mouse in which adenylyl cyclase type 8 (AC8) is overexpressed only in the heart (TGAC8), a model of accelerated cardiac aging [25]. Fourteen month-old TGAC8 (*n* = 8) and their wild-type (WT) littermates (*n* = 8) were treated with daily exposure to near-infrared light (850 nm) at 25 mW/cm^2^ for 2 min each weekday for a total dose of 1 Einstein (4.5 p.J/cm^2^ or fluence 3 J/cm^2^) and compared to untreated controls over an 8-month period. PBM therapy was administered for 3.5 months (early treatment period), paused, due to COVID‐19 restrictions for the following 3 months, and restarted again for 1.5 months. Serial echocardiography and gait analyses were performed at monthly intervals, and serum TGF-β1 levels were assessed following sacrifice. PBM treatment mitigated age-associated cardiovascular remodeling and reduced cardiac function, improved neuromuscular coordination, and increased longevity in TGAC8. These responses also correlated with increased TGF-β1 in circulation. Of note, most of the PBM effects measured at 3 months persisted 3 months following the resumption of the study after the COVID pause.

### Peripheral arterial disease

Several attendees also discussed PBM effects on peripheral arterial disease (PAD). This morbid condition, whereby ischemic peripheral muscle causes pain and tissue breakdown, affects 8–10 million Americans with a prevalence of ~ 20% among those individuals over 65 years of age. Notably, patients diagnosed with PAD have a 3–6 times increased likelihood of experiencing a lethal stroke or myocardial infarction. Currently, medical treatments are limited, with many patients requiring invasive procedures to address the impaired blood flow. A primary characteristic of PAD is endothelial dysfunction, which involves abnormal signaling and production of NO, resulting in microcirculatory dysfunction and reduced arterial and vasodilation. Accumulating evidence strongly indicates that dysregulation of the mechanisms involved in the generation of NO and ROS is an important cause of cardiovascular disease and a target for therapy [26]. Notably, when endothelial tissue has normal oxygen levels (normoxia), NO is produced via nitric oxide synthase (NOS) enzymes and ultimately stimulates vasodilation in the smooth muscle. However, in states of endothelial dysfunction with deficient oxygen (hypoxia) NOS activity is impaired, limiting the production of NO [26]. The body must then compensate for low oxygen levels, commonly seen in conditions of ischemia, by using a NOS independent system to produce NO by reducing nitrite [27].

Pursuing these ideas of the involvement of NO in arterial function, Lohr and colleagues found that deoxyhemoglobin possesses nitrite reductase activity, subsequently describing the mechanisms for S-nitrosothiol formation and degradation as mediated by visible light (340–545 nm). Moreover, NIR light (670 nm) was shown to act on deoxyhemoglobin to release NO, and this released NO enhanced the cardioprotective effects of nitrite [28].

Extending the work to an ex vivo murine model, Lohr and colleagues observed that NIR significantly increased vasodilation in wild-type and NOS inhibited groups, whereas NIR dilation was totally abolished with an NO scavenger or blood vessel denudation. The collective research revealed that light-dependent vasodilation is endothelial- and NO-dependent, but not endothelial NOS-dependent. Investigations of the bath solutions from NIR-treated vessels uncovered the presence of S-nitrosothiols, which were abolished in samples that underwent endothelial denudation [29]. Subsequent studies confirmed that a 670-nm light stimulates vasodilator release from an endothelial store, and that this paracrine-like substance has the characteristics of an S-nitrosothiol [30]. NIR light has also been demonstrated to increase expression of endosome associated membrane protein CD63 in bovine aortic endothelial cells and induce release of S-nitrosothiol containing extracellular vesicles from murine facialis arteries [31]. More recent studies measuring blood flow with laser Doppler perfusion imaging in mice have found that the 670-nm light can regulate dilation of the conduit vessel by releasing a vasoactive NO precursor species, presumably S-nitrosothiols [32]. By targeting these alternative sources of NO, NIR therapy may overcome NOS dysfunction in peripheral vascular pathologies, such as PAD, for the improvement of vascular health.

Ongoing studies using contrast enhanced ultrasound have revealed that the 670-nm light increases blood flow in healthy controls and PAD subjects. Current efforts are aimed at measuring S-nitrosothiols, a goal that has proven difficult in humans. Additional studies involving a PAD randomized trial, which is ongoing, have found that one 10-min NIR exposure increased the 6-min walk distance as well as walking pace. Future investigations targeting a range of clinical disorders that incorporate PBM therapy are underway or in the planning stage.

### Retinal disease

Given that the highest concentration of photoreceptors is in the eye, the Eells laboratory reviewed their 20 years of exploring the therapeutic benefits of PBM in retinal aging and disease. The fundamental organization of the retina is conserved across species and is comprised of five major neuronal classes (i.e., photoreceptors, bipolar cells, amacrine cells, horizontal cells, and ganglion cells), with Muller glial cells and retinal pigment epithelial cells providing metabolic and homeostatic support. Exposure to light initiates core signaling mechanisms in photoreceptors and consequent network responses that convert light energy into neural activity. Since the retina is one of the highest oxygen-consuming organs in the human body and reliant on mitochondrial function, dysfunction in this organelle and associated oxidative stress play a key role in retinal cell loss during aging and disease. Indeed, mitochondrial dysfunction and oxidative damage have been tightly associated with retinitis pigmentosa, glaucoma, age-related macular degeneration (AMD), diabetic retinopathy, and methanol intoxication [33]. As PBM has been shown to elicit its effects in part by targeting CcO and thereby mitochondrial activity and performance, Eells and colleagues have investigated its beneficial effects in a variety of disease and aging models.

Early studies into the effects of PBM used a rat model that involved methanol intoxication, which produces toxic injury to the retina and optic nerve by disrupting mitochondrial morphology and function, resulting in blindness [34]. In particular, formic acid, the toxic metabolite of methanol, is a reversible inhibitor of CcO (complex IV). Experiments demonstrated that three brief (2 min, 24 s) 670 nm LED treatments (4 J/cm^2^), delivered at 5-, 25-, and 50-h post-methanol intoxication, attenuated the retinotoxic effects of methanol-derived formate in rats [35]. Additionally, PBM therapy (830 nm NIR) administered during the critical period preserves mitochondrial redox state, mitochondrial function, oxidative homeostasis, and retinal responsiveness and protects against photoreceptor loss in a rat model of retinitis pigmentosa, harboring an autosomal dominant pathologic rhodopsin gene mutation (P23H) [36]. Using an in vitro model involving rat Müller glial cells grown under normal (5 mM) or high (25 mM) glucose conditions that replicates aspects of early diabetic retinopathy (DR), a single 670-nm light treatment diminished ROS production and preserved mitochondrial integrity [37]. PBM treatment for 3 days in culture reduced NFκB activity to levels observed in normal glucose and prevented the subsequent increase in ICAM-1, early molecular changes seen in DR. Conducting a small randomized clinical study on patients with diabetic macular edema, Eells and collaborators found that the 670-nm PBM therapy improves visual acuity and decreases retinal edema [38]. Moreover, three prospective, double-masked, randomized, multi-center clinical trials utilizing multi-wavelength treatment (590, 660, and 850 nm) of dry AMD subjects have documented improved visual acuity and contrast sensitivity and decreased drusen volume, suggesting widespread applications for PBM [39].

### Parkinson’s disease

Participants also noted the potential for PBM in treating Parkinson’s disease (PD). Recent investigations by Mitrofanis and colleagues has uncovered that NIR light (670 nm) can reduce Parkinson-like phenotypes, including both behavioral and structural measures, of MPTP-treated rodents and primates [40]. Spurred on by these observations, follow-up studies found that PD patients treated with NIR light, either via a helmet (a.k.a. bucket) or intranasal device, exhibited improved (55%) or no change (43%) in the initial signs and symptoms of the disease, supporting future clinical research in the area of PBM and neurogenerative disease [41].

NIR light application in Parkinsonian MPTP animal models also appears to help preserve dopaminergic neurons in STN (substantia nigra) from MPTP induced degeneration and locomotor activity [42]. While numerous animal models of PD have been studied [43], large-scale human RCTs have yet to be completed. However, preliminary data in humans suggest that PBM can improve measures of mobility, cognition, balance, and motor skill for at least 12 weeks after treatment [44, 45]. In addition, neurosurgeon Alim-Louis Benabid of the Clinatec Institute is overseeing a large clinical trial with patients with early stage PD where they will deliver a 670-nm light intracranially over 4 years [7].

### Brain networks and cognition

**Fig. 5:**
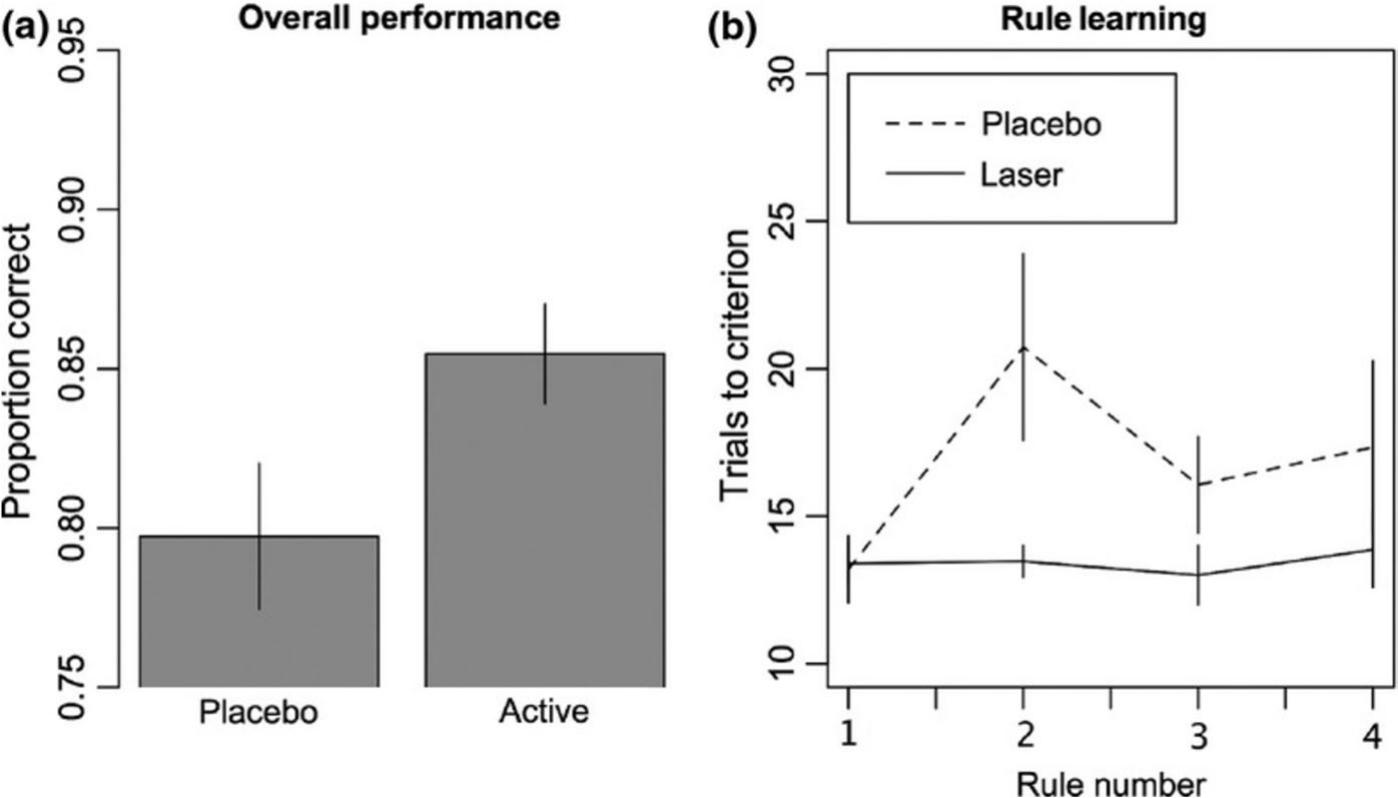
Overall accuracy on the Wisconsin Card Sort Task across all trials for the two groups. **a** The active PBM laser treatment group correctly sorted the cards more often than the placebo group. **b** Trials to criterion for each of the first four rules learned. The placebo treatment group took significantly longer to reach criterion on the second rule than the active laser treatment group, suggesting a benefit in set‐shifting ability in the active treatment group. Error bars represent standard errors. Reproduced from Blanco et al. (2017)

Discussion of the extent to which PBM affects neural circuitry and cognition was also compelling. Dr. Gonzalez Lima reviewed investigations conducted to evaluate the effects of PBM on physiological parameters including brain electroencephalogram (EEG) rhythms. In studies involving human subjects, transcranial PBM via a 1064-nm laser was found to significantly increase resting-state EEG spectral powers at both the alpha (8 to 13 Hz) and beta (13 to 30 Hz) bands and to promote a more efficient prefrontal blood-oxygen-level-dependent functional magnetic resonance imaging response [46]. Based on 3-D brain maps, PBM also modulates a large scale fronto-parieto-occipital network involved in attention, memory, and executive functions, and improves cerebral blood flow even beyond the site of treatment [47, 48]. Additionally, participants who received the 1064-nm laser treatment made fewer errors and showed improved rule learning in the Wisconsin card sorting task relative to placebo controls, indicative of improved executive function (Fig. 5; [49]). Examining the effects of PBM on attention and working memory tasks in older individuals (between 49 and 90 years of age), 5-week sessions of PBM were also found to improve acute and chronic cognitive function [50]. As brain physiology is critically dependent on oxygenation for energy production and network function, these mechanistic studies of PBM subjects suggest that PBM, which serves to enhance cognitive processing by the brain, may be a safe, non-invasive, and effective strategy for the treatment of neuropsychological disorders and normal aging.

## Utilizing rigorous and reproducible science

Looking to the future, it’s evident that PBM is relatively straightforward to administer but difficult to get right, primarily due to the non-linear responses to light exposure. This non-linearity, which is seen in other biological systems (e.g., photosynthesis), is known as the Arndt-Schultz law, where the optimal dose induces a therapeutic benefit, while too low of a dose has no effect and too high of a dose has a detrimental consequence [51]. Emphasis therefore needs to be placed on determining the right cellular/tissue context, considering cell heterogeneity, as well as the correct PBM therapy, which would encompass the delivery mechanism, wavelength, and dose. Towards optimizing the treatment protocols, studies in mice have pointed to the utility of monitoring surface temperature and ATF4 expression as negative biomarkers to identify safe and effective clinical PBM therapies [52]. Currently, PBM is recommended as standard of care for oral mucositis, a common side effect of radio or chemotherapy. Clinical guidelines are also now being developed for several other indications, including wound healing, regenerative medicine, dentistry, and neurorehabilitation, a step that will improve the rigor and reproducibility of PBM therapeutic responses going forward.

### Molecular mechanisms

One of the major discussion points centered around the need for a deeper understanding of the molecular mechanisms that underlie the therapeutic benefits of PBM seen in animal models and human trials. While there is growing consensus around the main targets of PBM, such as CcO, there remain significant concerns about the apparently conflicting results seen between simplified, reconstituted systems with purified enzymes and more complex in vivo models. In fact, PBM (660 nm) enhancement of cell proliferation has been reported not to require CcO in both mouse and human cell line models, implying alternative mechanisms are at play [53]. Whether the reported discrepancies arise due to technical issues, experimental parameters, or the complexity of biological systems requires clarification. There were also questions regarding the contribution of calcium signaling and epigenetics in the PBM response. While long-term epigenetic reprogramming has not been seen in the absence of chronic stimulation therapy, more precise and extensive answers on these topics await further investigation, some of which is ongoing. It was generally agreed among the participants that more rigorous, well-defined methods and approaches must be devised and implemented to definitively characterize the primary molecular targets and signaling pathways activated by PBM, potentially focusing an aspects of intrinsic resilience mechanisms.

### Dosing and application

Related to the above are issues associated wavelength and dosing, as well as the non-linear responses reported for PBM treatments. Since these features can often vary from study to study, understanding the underlying specificity of the different treatment paradigms (e.g., wavelength), in terms of mechanisms and effects, and developing subsequent standards for PBM therapy remains a priority. Moreover, it is critical to keep in mind the local, surrounding environment, depth of penetration needed, and cells being traversed when planning and anticipating the effects of PBM. Certainly, there will be cell-specific responses dictated by treatment protocol, intracellular protein content, and active signaling mechanisms. Although overtreating does not appear to cause adverse outcomes, it runs the risk of eliminating the beneficial effects of light therapy. Thus, the long-term vision must focus on establishing the “sweet spot” for a precise PBM therapy and generating standard of care guidelines, namely the dose estimate, specificity of the target and its surrounding features, as well as underlying mechanisms. How this goal should be achieved and what models should serve in standardizing the methods needs to be determined.

### Proximal vs. distal effects

It is known that the penetration of red light into the skin is relatively shallow (likely around 1 cm), raising the question of how systemic effects are induced by PBM therapy. Consistent with a paracrine-type effect of PBM, it has been reported that NIR therapy applied to the dorsum of an animal is protective, albeit less so than transcranial or intracranial treatments, against MPTP-induced neurodegeneration of the substantia nigra [54]. Similar distal PBM effects have been described elsewhere [55]. While it seems clear that NO plays some role in both proximal and distal responses, there are almost certainly other circulating mediators, such as peroxynitrate and additional ROS, which can participate in vasodilation and the other physiological effects. Identification of these systemic molecules is essential to understand the broader mechanisms of PBM and its clinical benefits. Another future point of emphasis could be focused on looking at integrating other sense modulators as well, in addition to preconditioning strategies, such as exercise regimes, diet, and supplements.

## Conclusion

While emerging acceptance of the health benefits of PBM therapy and beneficial results have been seen in people thus far, true clinical utility may not be realized until evidence-based treatment options are made more available to a wider number of patients. One obstacle pointed out in this process is the fact that, while there is significant financial motivation to identify novel pharmaceuticals, there is little monetary incentive to pursue optimal light treatments, as PBM treatments can be administered without specialized devices (red light and near-infrared light LEDs are available from numerous commercial sources). Despite these challenges, one company has conducted double-masked, randomized, multi-center clinical trials to develop and market PBM devices to treat macular degeneration [56]. These are the types of experimental trials necessary to advance PBM therapy into common clinical practice. For instance, while there is current interest in developing such trials around cancer, there remains the issue of the optimal therapeutic wavelength and dosing that must be resolved and agreed upon. As therapies become more advanced in their target specificity, reporting landmark observations in broader audience journals will be imperative to gain widespread understanding and support of the potential benefits of PBM.

## Data Availability

This manuscript reports data based on original published studies and the viewpoints of individual researchers who contributed to the workshop. The corresponding author, David Frankowski, may be contacted for further information.
